# Quality of a life skills training program in Karnataka, India - a quasi experimental study

**DOI:** 10.1186/s12889-019-6836-8

**Published:** 2019-05-02

**Authors:** Banandur S. Pradeep, Banavaram Anniappan Arvind, Srinivas Ramaiah, Swati Shahane, Lavanya Garady, Mutharaju Arelingaiah, Gopalkrishna Gururaj, Gananatha Shetty Yekkaru

**Affiliations:** 1 Department of Epidemiology, National Institute of Mental Health and Neurosciences, Bengaluru, 560029 India; 2 Administrative Medical Officer, Government Taluka Hospital, Gauribidanur, 561208 India; 3 Department of Youth Empowerment and Sports, Government of Karnataka, State Wing, National Services Scheme, Bengaluru, 560029 India

**Keywords:** Teachers, Life skills education, Training, Quality, India

## Abstract

**Background:**

Youth focused Life Skills Education and Counseling Services (YLSECS) program, trained teachers/National Service Scheme (NSS) officers to deliver Life Skills Education (LSE) and counseling services to college going youth in the state of Karnataka in India. Available evaluation of life-skills training program have neglected the recording and or reporting of outcome among those trained to implement life-skills training program. Present paper highlights the quality of YLSECS training program and change in perception among teachers/NSS-officers trained in-terms of improvement in their cognitive/affective domains.

**Methods:**

YLSECS program focused on World Health Organization identified ten essential domains of life-skills. Participants of the YLSECS program were trained by adopting facilitatory approach based on the principles of Kolb’s learning theory. Quasi experimental study design was used to evaluate the outcome of training among participants. Quality of the training was assessed using scoring system and change in perception was assessed using Likert scale. Statistical significance of change in perception before and after training was assessed by paired‘t’ test for proportion.

**Results:**

Overall, 792 participants rated the quality of training as either “good” or “excellent”. Post-training, significant (*p* < 0.001) proportion of the participants reported improved awareness about life-skills (before training 49.9 to 74.4% vs post-training range from 91.6 to 95.1% for various domains). There was statistically significant (p < 0.001) increase in participants reporting “very confident” in teaching various life skill domains (before training from 22.7 to 34.2% for various domains and post-training it ranged from 65.2 to 74.7% for various domains). There was modest increase in participants reporting perceived ability to conduct life-skills workshop “without assistance” post-training (before training from 16.8 to 22.9% for various domains vs post-training ranged from 29.8 to 36.8% for various domains). Interestingly, considerable proportion of participants who prior to training reported being confident in providing life skills training (without any assistance), later (i.e post training) reported they need some/more assistance for the same.

**Conclusion:**

YLSECS training program significantly improved participants knowledge and confidence in imparting life-skills and highlight the need for continued handholding of participants for effective implementation of LSE and counseling service program.

## Background

The demographic-socioeconomic-political development of a nation is dependent on its youth population. “Youth bulge”, in India presents an opportunity to harness the demographic dividend [1]. To maximize the benefits of this demographic dividend, youth need to be healthy, educated and skilled [2]. But conversely significant proportion of youth in India is affected with several health problems including poor mental health [3–5]. Therefore, empowering youth with quality education and twenty-first century skills like creative thinking, decision making, communication etc. would help them to remain healthy and gain employment [3, 6], thereby enable youth to contribute productively to the society. Unfortunately, the current system of education in the country is restricted in its ability to provide youth with these necessary skills [7] with a similar situation in Karnataka (state in southwestern region of India).

Globally preparing youth to thrive in the future knowledge economy, in the light of advancements in technologies and industries, is considered a major challenge for the twenty-first century [8]. Life Skills Based Education is known to play a crucial role in youth health promotion, education and overall development [9] by enabling them to adopt healthy personal behaviors, improving educational outcomes and earning potential [10, 11]. Considering the need, National Service Scheme wing, Department of Youth Empowerment and Sports, Government of Karnataka supported by Department of Epidemiology, Centre for Public Health, National Institute of Mental Health and Neuro Science (NIMHANS), initiated the “Youth focused Life Skills Education and Counseling Services (YLSECS) program” for promoting mental health and well being of youth in the state.

The National Service Scheme (NSS) is a Scheme of Government of India, Ministry of Youth Affairs & Sports, which aims at providing an opportunity to the student youth of India to take part in various government led community service activities and programs [12]. YLSECS program, therefore, focused on training NSS coordinators/officers and teaching faculty from the departments of pre-university education, collegiate education, technical education and medical education. Training was aimed at enhancing and strengthening the knowledge and capacity of NSS coordinators/officers and teachers to impart life skills training to youth in their respective institutions. Review of life skills education programme implemented across the globe, has observed that existing training under such programme does not adequately address and report the psychosocial skills and attitudes of teachers (who are expected to train students) trained [10] The Present paper addresses this information gap by reporting the quality, of YLSECS training program and post training improvement in knowledge and confidence among participants (including NSS coordinators/officers and teachers) to impart life skills training.

## Methods

Between January and July 2017, 28 life skills training workshops were conducted under YLSECS program. These trainings were conducted in a class room setting in the department of Epidemiology at NIMHANS, Bangalore. Participants (NSS officers/coordinators and teachers) for the training were deputed by the government agencies. The entire training program was conceptualized around adult learning principles emphasizing active participatory teaching-learning methods. Ten different domains of Life skills as identified by World Health Organization [13] and expert consultation namely: self-awareness, empathy, coping with stress, coping with emotions, interpersonal skills, critical thinking, decision making, problem solving, communication skills and creative thinking were selected for training under the YLSECS program. The training program served the dual purpose of equipping participants with relevant life skills knowledge and also to demonstrate how to conduct such trainings in their respective settings through facilitatory approach.

### YLSECS training process

Overall, each training workshop was planned for 5 days and each day 2 domains were covered involving 3–4 h per domain. In each workshop 25 to 30 participants were enrolled and they were trained by master facilitators. Facilitators were specialist in the field of psychology and social sciences and were skilled in conducting the training utilizing facilitatory approach.

The process of training participants in YLSECS program was grounded in Kolb’s learning theory which emphasizes learning as the process whereby knowledge is created through the transformation of experience [14]. Accordingly, the process of facilitation in a particular domain of life skills involved sequential steps namely activities, reflective observation, abstract conceptualization and summarizing. Firstly, trainees participated in an activity which was designed to expose the participants or make them experience a situation that needs them to use a particular life skills domain. Subsequently, participants were asked to reflect on their perception, experience and opinion about the activity (Reflective Observation). This was followed by Abstract Conceptualization, wherein facilitator conceptualized the activity performed underpinning the role of a particular Life Skill domain. This enhances participants understanding and knowledge about that particular life skill domain. Towards the end, each session was concluded by summarizing the learnings together with suggestions for the trainers on how to effectively implement the activity/training in their respective institutions.

Predesigned semi structured self administered feedback evaluation questionnaire was developed to collect information on sociodemographic characteristics of the participants, their feedback on quality of each session of training and the outcome of YLSECS training program. Effect of the training in improving participant’s awareness about life skills, increasing their level of confidence in teaching life skills and perceived ability to independently conduct life skills training workshop was assessed through a quasi experimental study -the one group pretest posttest study design. Information was collected from the participants immediately before and after the training program through trained data collectors.

Quality of the training was assessed considering the content, communication, presentation skills and teaching methodology adopted by the facilitator. For each of the component participants scored on a 5-point scale: Poor (score – 0), Average (score – 1), Good (score – 2), Very good (score – 3) and Excellent (score – 4). The total score for each domain was calculated by summing the scores of all components. Thus, for each component the score ranged between 0 and 4 and for each life skill domain the score ranged between 0 and 16. Based on the total score, quality of the training was graded as follows: poor (Scores – 0 to 4), fair (score- 5 to 9), good (score- 10 to 13) and excellent (score- 14 to 16).

Effect of training program in improving knowledge and perception was assessed by considering the following variables among participants viz. awareness regarding life skills, level of confidence in teaching life skills and level of perceived ability to independently conduct life skills training program. Awareness about life skills was assessed through questions that elicited binary response (Yes or No) for various domains of life skills. Participants ‘Level of Confidence’ in teaching Life skills and ‘Perceived Ability’ to independently conduct the Life skills training program was assessed using likert scale. “Level of confidence” was graded as not confident, somewhat confident and very confident and “perceived ability” was graded as without assistance, with little assistance and with more assistance. Each participant was given a unique training ID number to pair his/her given information before and after the training program.

The difference in the responses regarding awareness of life skills, level of confidence to teach life skills and perceived level of assistance needed for independently conducting Life Skills training program before and after the training was assessed by paired “t” test for proportion. All analysis was undertaken using SPSS version 22.0 and *p*-value < 0.05 was considered statistically significant.

## Results

Over a period of 7 months, 792 participants were trained under YLSECS program. Mean age of the participants was 40.1 years and males constituted 78%. Majority of the participants had completed diploma/degree/ postgraduate studies (98%) and were employed either as lecturer (42.7%) or as assistant professor (32.3%) in their respective institutions (Table 1).

**Table 1:** Socio demographic characteristics of participants of YLSECS training program

Socio demographic characteristics (*n* = 792)	N (%)
Age Mean (SD)	40.1 (9.7)
Gender	
Male	615 (78.0)
Female	177 (22.0)
Place of residence	
Urban	443(56.0)
Rural	349 (44.0)
Education (highest standard studied)	
High school	3 (0.4)
Pre-University (Class 11 and 12)	13 (1.6)
Degree/Diploma	152 (19.2)
Post-graduation and above	624 (78.8)
Current position	
Lecturer	338 (42.7)
Assistant Professor	256 (32.3)
Others	37 (4.7)
YPs	161 (20.3)
Religion	
Hindu	729 (92.0)
Others	63 (8.0)
Marital status	
Currently married	623 (78.1)
Never Married	153 (19.3)
Others	16 (2.0)

**Fig. 1:**
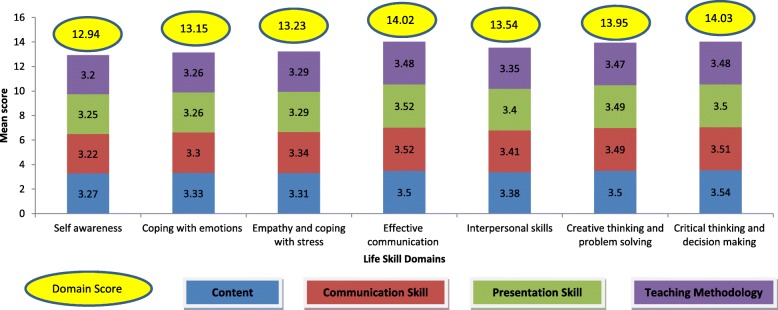
Feedback of participants on domain and component wise quality of YLSECS training program. For each domain (i.e self-awareness, coping with emotion etc), score ranges between 0 and 16; For each component within a given domain (i.e content, communication skill, presentation skill, teaching methodology), score ranges between 0 and 4

Overall, the quality of training for various domains of YLSECS training program was rated as either “Good” or “Excellent”. The quality of training for effective communication (mean score = 14.02) and critical thinking/decision making (mean score = 14.03) was rated as excellent. The content, communication, presentation and teaching methodology for all the domains was rated as “Very Good (score-3)” or “Excellent (score-4)” (Fig. 1).

**Fig. 2:**
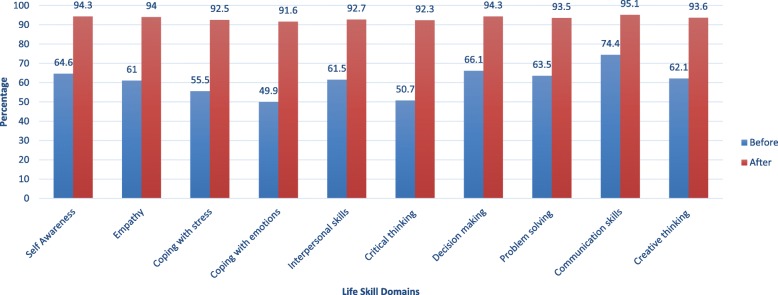
Percentage of participants who were aware about 10 domains of Life Skill before and after training

There was statistically significant (*p* < 0.001) improvement in awareness about different domains of life skills after training. Before training, awareness about different domains of life skill varied between 49.9% (for coping with emotions) to 74.4% (for communication skills). After training, for each domain, more than 90% of the participants reported improved awareness. The percentage increase in awareness was relatively more in the domains of coping with emotions (41.7%), critical thinking (41.7%) and coping with stress (37.0%) (Fig. 2).

The proportion of participants in “very confident” category under various domains of life skills before training ranged from 22.7% for coping with emotion to 34.3% for communication skills and after training it varied from 65.2% for coping with stress to 74.7% for communication skills (i.e more than doubled across all domains of life skills). This difference in proportion was statistically significant (*p* < 0.05) (Table 2). Similarly, there was statistically significant (p < 0.05) increase in proportion of participants reporting ability to conduct life skills workshop without any assistance after training (ranged from 29.8% for critical thinking to 36.8% for communication skills) (Table 3). Overall, after training, the number of participants who felt they could impart life skills training without any assistance increased by one and half to two times across all the domains.

**Table 2:** Perceived level of confidence in teaching Life Skills among participants before and after training

Sl no	Life skills	Before training			After training			‘*p*’ value
		Not confident n (%)	Somewhat confident n (%)	Very confident n (%)	Not confident n (%)	Somewhat confident n (%)	Very confident n (%)	
1	Self-awareness	141(18.2)	437(56.5)	196(25.3)	15(1.9)	217(28.1)	539(69.9)	< 0.0001
2	Empathy	182(22.7)	386(50.3)	199(25.9)	21(2.7)	217(28.2)	532(69.1)	< 0.0001
3	Coping with stress	206(26.7)	379(49.2)	186(24.1)	29(3.8)	238(31.0)	500(65.2)	< 0.0001
4	Coping with emotions	210(27.7)	375(49.5)	172(22.7)	28(3.6)	236(30.7)	504(65.6)	< 0.0001
5	Interpersonal skills	178(23.5)	387(51.1)	193(25.5)	15(2.0)	236(30.7)	517(67.3)	< 0.0001
6	Critical thinking	220(29.3)	348(46.4)	182(24.3)	22(2.9)	239(31.3)	503(65.8)	< 0.0001
7	Decision making	148(19.4)	394(51.7)	220(28.9)	17(2.2)	208(27.0)	544(70.7)	< 0.0001
8	Problem solving	164(21.9)	350(46.7)	236(31.5)	16(2.1)	201(26.3)	548(71.6)	< 0.0001
9	Communication skills	125(16.4)	376(49.3)	261(34.3)	18(2.3)	176(22.9)	574(74.7)	< 0.0001
10	Creative thinking	188(23.9)	366(46.6)	212(27.0)	17(2.2)	227(29.7)	520(68.1)	< 0.0001

**Table 3:** Perceived level of assistance needed in conducting Life Skills workshops among participants before and after training

Sl no	Life skills	Before training			After training			*p* value
		With little assistance n (%)	With more assistance n (%)	Without assistance n (%)	With little assistance n (%)	With more assistance n (%)	Without assistance n (%)	
1	Self-awareness	405(52.8)	233(30.4)	129(16.8)	341(44.2)	159(20.6)	271(35.1)	< 0.0001
2	Empathy	352(46.5)	263(37.7)	142(18.8)	359(46.7)	150(19.5)	260(33.8)	< 0.0001
3	Coping with stress	365(48.3)	240(31.7)	151(20.0)	366(47.5)	156(20.3)	248(32.2)	< 0.0001
4	Coping with emotions	372(49.5)	241(32.1)	138(18.4)	367(47.8)	159(20.7)	242(31.5)	< 0.0001
5	Interpersonal skills	373(49.8)	232(31.0)	144(19.2)	359(46.6)	162(21.0)	249(32.3)	< 0.0001
6	Critical thinking	337(45.3)	255(34.3)	152(20.4)	360(47.1)	176(23.0)	228(29.8)	< 0.0001
7	Decision making	352(46.9)	247(32.9)	151(20.1)	337(44.1)	164(21.5)	263(34.4)	< 0.0001
8	Problem solving	331(44.3)	264(35.3)	152(20.3)	359(47.0)	157(20.5)	248(32.5)	< 0.0001
9	Communication skills	340(45.3)	239(31.8)	172(22.9)	309(40.1)	178(23.1)	283(36.8)	< 0.0001
10	Creative thinking	320(42.6)	266(35.4)	165(22.0)	369(47.7)	172(22.3)	232(30.0)	< 0.0001

Among those participants who reported to be not confident of conducting a life skills training program before training, nearly 90% of the participants shifted to either somewhat confident or very confident category after training (range – 91.6% for self awareness to 96.7% for problem solving). Similarly, among those participants who belonged to somewhat confident category before training, more than 2/3rd of them (range – 57.4% for coping with emotions to 70.8% for communication skills) moved to very confident category after training. Majority (ranging from 82.3% for critical thinking to 89.5% for communication skills) of the participants who expressed they were very confident before the training program remained very confident after the training program (Table 4).

**Table 4:**
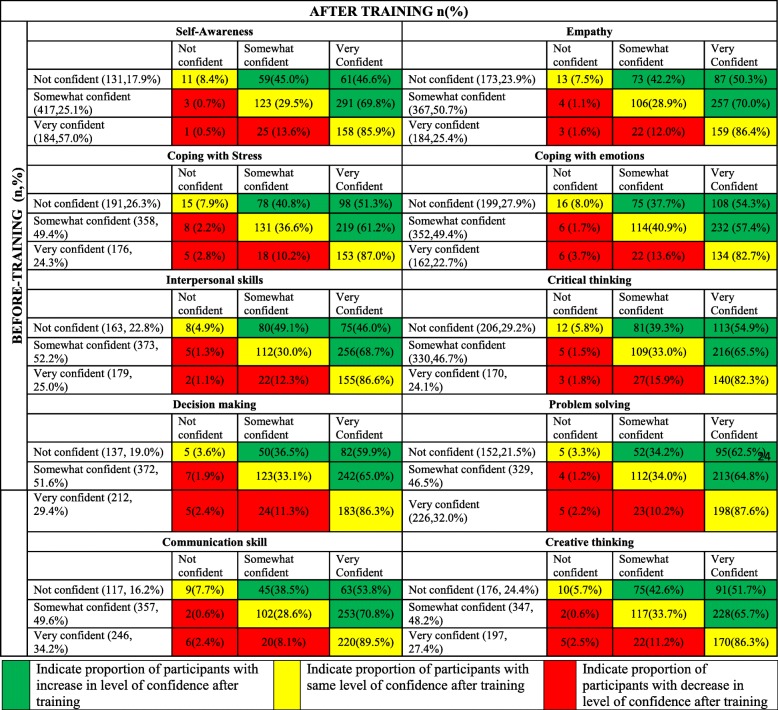
Movement of participants between different categories of perceived level of confidence in teaching Life Skills (for each domain of life skills) after training

In terms of the perceived ability to conduct the life skills training program with or without assistance (Table 5), among the participants who had thought they would need more assistance before the training program, majority of the participants reported improved ability after training. Nearly 2/3rd to 3/4th of the participants opined they would need less or no assistance to conduct the life skills training program after undergoing the training (Range – 67.2% for communication skills to 80.2% for Empathy). Among the participants who expressed the need for less assistance before training, nearly 1/3rd of the participants reported they do not need assistance after the training (Range – 29.4% for creative thinking to 38.1% for self-awareness and Communication skills) and nearly 1/5th of them reported that they need more assistance after-training (range 17.8% for creative thinking to 20.4% for critical thinking).Among those who reported that they do not require any assistance prior to training, nearly 50% reported that they either need little or more assistance after the training (range – 40.5% for communication skills to 54.8% for coping with stress).

**Table 5:**
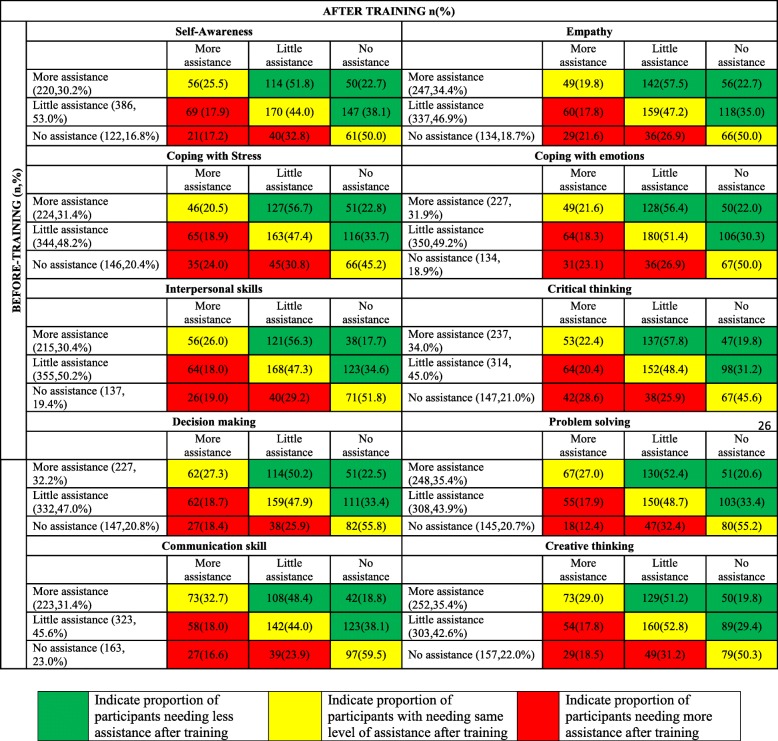
Movement of participants between different categories of perceived level of assistance needed in conducting Life Skills workshops (for each domain of life skills) after training

## Discussion

YLSECS training program is perhaps the largest training program, in the state of Karnataka, aimed at empowering NSS officers and other teaching faculty with requisite knowledge and skills for imparting life skills to college going youth. The National Service Scheme **(**NSS) implemented in educational institutions of the state, through NSS officers, work towards developing the personality and character of the student youth through voluntary community service [7]. In this context, training of NSS officers and teachers who can reach considerable segment of youth population was deemed appropriate which ensured adequate support by government and educational institutions contributing to successful implementation of YLSECS program.

A systematic and objective assessment of the quality of YLSECS training program and its effect on participants knowledge and perception was undertaken and it was observed that nearly 90% of the participants rated the quality of training as either “Very Good” or “Excellent” (data not shown) which inturn is reflected in the domain mean scores (Fig. 1). Training has significantly improved participant’s awareness about life-skills (level of awareness post training ranged between 91.5% for coping with emotion to 95% for communication skills) and increased their level of confidence in teaching life skills to students (percentage of participants reporting “very confident” ranged between 65% for coping with stress to 75% for communication skills post training). Considerable improvement in level of awareness was observed in the domains of coping with stress, coping with emotions and critical thinking. Interestingly, considerable proportion of participants who prior to training reported being confident in providing life skills training (without any assistance), later (i.e post training) reported they need some/more assistance for the same. It is likely that the participants, post training, would have possibly understood and got grounded about the nuances of life skills and more importantly the facilitatory role they need to play.

The successful implementation of life skills education programme depends upon the provider confidence (one who imparts or conducts life skills training) and on the continued support and regular refresher training he/she (provider) receives [13, 15]. Though considerable proportion of participants, in this study, expressed that they were confident in teaching life skills, only 30–37% of the participants, post training, perceived that they could independently conduct life skills training workshop without any (technical) assistance. This probably indicates that participants could be confident in teaching life skills in one-to-one basis, but not confident in conducting such training for large number of people in workshop mode. However, this is a natural process of learning and participants would feel confident in conducting life skills training workshop independently only upon implementing the training in their respective institutions and through the experience gained thereof. Furthermore, this describes the need for hand holding and supportive supervision for participants to conduct life skills training in their respective colleges. Therefore, as part of the program, following training, each participant is mandated to train 500 youth on life skills in their workplace. The YLSECS program team will provide hand-holding and supportive supervision to the teachers during their training workshops.

Active participatory learning is central to imparting life skills [16] and is considered the basis for training of life skills trainers [13, 17, 18]. YLSECS training program was strongly grounded on facilitation cum participatory approach to training and was designed based on adult learning principles. This ensured active participation and learning by the participants which is probably reflected in the outcome of training. Furthermore, all the trainings were facilitated by experienced life skill training experts (psychologist, sociologist and others) who were well versed in training utilizing facilitation methods. Facilitation is known to provide experiential learning especially when learning from groups is required or when situation/question do not have a simple right answer and learning from others experience and ideas is required [19]. Life skills fit this criterion very well and hence the adoption of facilitation as a mode of imparting life skills to this group was envisaged.

Good quality training improves the professional skills and competencies needed for imparting life skills education among teachers [15, 20, 21]. Improving teacher’s skills in this regard is of high priority in Life skills education programme being implemented in educational institutions [22]. The impact of skill based training is critically dependent on the facilitator (one who provides training) and most of the life skills education programme evaluation [10, 23, 24] especially in which teachers are trained to impart life skills education to students, do not provide information on quality of the training and outcome among the teachers trained. The present study, conducted in a low resource setting, provides information to fill this critical knowledge gap.

Despite demonstrating significant improvement in knowledge, confidence and ability to conduct life skills training among the participants, present study has some limitations. Firstly, participants knowledge and perceived ability was assessed immediately after training and hence they do not reflect the sustained improvement in the outcome. However, there is an inbuilt evaluation planned under YLSECS program with a comparison group at three time points within a period of 1 year post training. Secondly, assessment of confidence and perceived ability to conduct life skills training was assessed using self administered questionnaire. It would have been ideal to assess such parameters by observing the participants while they are actually conducting life skills training. This is also being addressed as part of continued evaluation of YLSECS program by observing the teachers providing life skills training to their students or community in their own setting. Thirdly, we didn’t assess the effect of facilitation as a mode of delivery, as this requires comparison with other methods of training (ex involving didactic method), due to resource constraints. However, feedback forms received from participants provides anecdotal evidence on the effectiveness of facilitation methods (data not shown). Finally, the participants being teachers were well educated with adequate teaching experience with many of them being NSS officers, they might have prior experience of life skills or maybe they are better implementers. Hence their understanding and acceptance about life skills would be better. The effect of such selection bias, if it exists, may not be ruled out in this study. Therefore, the results need testing if the training involves general population or other population groups.

## Conclusion

To conclude, the YLSECS training program has significantly improved participant’s knowledge and confidence about life skills and in implementing life skills training. However, there was modest increase in percentage of participants who perceived that they could implement life skills training without assistance. Based on the empirical evidence, it is strongly recommended that continued support and regular refresher training of teachers (or participants) who have undergone life skills training should be an essential component of life skills education programme. Furthermore, results of the study indirectly indicate the effectiveness of facilitating the process of learning among adult learners using active participatory approach and such or similar methods should be adopted for implementing life skills training among youth. The Karnataka state youth policy (2012) [25] and National Youth Policy of India (2014) [26] has identified life skills approach as one of the priority intervention for overall development of youth**.** Utilizing this opportunity, systems should be developed wherein resource persons/facilities skilled in life skills training, could be networked and tasked with providing onsite and regular refresher training of NSS officers/teachers and continuously monitor and evaluate such trainings.

## Abbreviations

NIMHANS: National Institute of Mental health and Neuro Sciences
NSS: National Service Scheme
YLSECS: Youth focused Life Skills Educations and Counseling Services
